# Mathematical modeling of the lower urinary tract: A review

**DOI:** 10.1002/nau.24995

**Published:** 2022-06-26

**Authors:** Daniel Jaskowak, Roberto Nunez, Rahul Ramachandran, Elie Alhajjar, John Yin, Giovanna Guidoboni, Zachary C. Danziger

**Affiliations:** 1 Department of Biomedical Engineering, Florida International University, Miami, Florida, USA; 2 Department of Electrical Engineering and Computer Science, University of Missouri, Columbia, Missouri, USA; 3 Department of Chemical and Biological Engineering, Wisconsin Institute for Discovery, University of Wisconsin–Madison, Madison, Wisconsin, USA; 4 Department of Mathematical Sciences, United States Military Academy, New York, New York, USA; 5 Department of Mathematics, University of Missouri, Columbia, Missouri, USA

**Keywords:** bladder biomechanics, computational model, modularity and interoperability, multiscale modeling, neurourology, predictive medicine, systems physiology

## Abstract

**Aims:**

Understand what progress has been made toward a functionally predictive lower urinary tract (LUT) model, identify knowledge gaps, and develop from them a path forward.

**Methods:**

We surveyed prominent mathematical models of the basic LUT components (bladder, urethra, and their neural control) and categorized the common modeling strategies and theoretical assumptions associated with each component. Given that LUT function emerges from the interaction of these components, we emphasized attempts to model their connections, and highlighted unmodeled aspects of LUT function.

**Results:**

There is currently no satisfactory model of the LUT in its entirety that can predict its function in response to disease, treatment, or other perturbations. In particular, there is a lack of physiologically based mathematical descriptions of the neural control of the LUT.

**Conclusions:**

Based on our survey of the work to date, a potential path to a predictive LUT model is a modular effort in which models are initially built of individual tissue-level components using methods that are extensible and interoperable, allowing them to be connected and tested in a common framework. A modular approach will allow the larger goal of a comprehensive LUT model to be in sight while keeping individual efforts manageable, ensure new models can straightforwardly build on prior research, respect potential interactions between components, and incentivize efforts to model absent components. Using a modular framework and developing models based on physiological principles, to create a functionally predictive model is a challenge that the field is ready to undertake.

## 1 | INTRODUCTION

It is difficult to make precise predictions about lower urinary tract (LUT) function because its behavior arises from the complex interactions of many tissues and organs spanning both space (from the bladder to the pelvic floor to the spinal cord) and time (from minutes of slow bladder filling to seconds of dynamic voiding). Mathematical models of physiological systems can characterize these multiscale interactions which enable us to make otherwise impossible quantitative predictions about how diseases or therapies affect their function through computational simulation. Currently, there is no functionally predictive mathematical model of the LUT, and without it, our only recourse to deepen our knowledge of its behavior and prototype new therapies is through slow in vivo experimentation. With an estimated 60% of adults developing at least one lower urinary tract symptom (LUTS) by age 40,^1,2^ a mathemat ical LUT model would greatly improve our ability to explore and design modern treatments to this large and growing public health issue. The goal of this review is to synthesize the body of LUT modeling efforts, highlight modeling attempts to predict clinically relevant phenomena, and provide a perspective on how to move toward creating a functionally predictive model of the LUT.

A mathematical model can serve as a virtual laboratory that complements observations from experimental data and can provide insight into the mechanistic function of organ systems or potential therapeutic strategies.^3,4^ For example, therapeutic strategies for neuromodulation have been found without a clear understanding of their mechanisms of action,^5^ leading to speculation that we could design more effective versions if we understood how the mechanisms worked at a fundamental level. A mathematical model that captures the function of the LUT could be used to systematically evaluate simulations of different neuromodulatory parameters (e.g., locations, frequencies, pulse trains, etc.) that would identify the most effective methods of treatment. The results of such in silico experiments could then lead to more focused in vivo experimentation to validate the results of the simulations and help clarify its mechanisms of action.

To coordinate efforts in the development of a model of LUT function, the state of LUT modeling must be compiled and evaluated so that gaps can be identified and targeted by future modeling efforts. Here we will discuss the common strategies for modeling the three key components of the LUT (bladder, urethra, and neural control). The bladder and urethra have adequate representations that reflect the biomechanics and fluid dynamics of the LUT, but available models of the neural control are rather insufficient, which is largely due to difficulty discerning corresponding neural activation to variations in LUT biomechanics and fluid dynamics. In our review of the literature, we compiled the prominent models that we believe facilitate the conception of a fully predictive model. In the chord diagram of Figure 5, these models are represented in terms of the basic LUT components and the interactions between them.

Given that the key LUT organs span multiple spatial scales and have complex interdependencies, we expect that the development of a model of LUT function will be a modular process in which models of individual LUT components (bladder, urethra, and neural control), possibly developed by different research groups, will be connected to form a comprehensive model.^6^ From a technical standpoint, the greatest difficulty might not be the creation of models of individual components, but rather the connections between such models. An appropriate choice of model structure could facilitate the production of these connections.

Most existing mathematical models of the LUT can roughly be classified in two model structures: *lumped models* and *spatio-temporal models*. Lumped models neglect spatial differences and instead focus on quantities that change with time. For example, although there are regions of low and high fluid velocity inside the bladder during voiding,^7,8^ many LUT models disregard them and consider only the net flow in and out of the bladder. As a result of these simplifying assumptions, the mathematical formulation of lumped models tends to be relatively simple and is amenable for analytical and numerical studies. Lumped models usually consist of ordinary differential equations (ODEs) that describe the time rate of change in physiological quantities. Spatio-temporal models, by contrast, do incorporate this spatial variation, which comes at the cost of increased mathematical complexity, which makes the analytic and numerical study of the model more challenging. Given that the connections between LUT components are quite complex, the mathematical simplicity of lumped models makes them a good starting point. However, it is unlikely that lumped models will be able to accurately describe all aspects of the behavior of the LUT (especially those influenced substantially by spatial variations). A base lumped model can then be enhanced by incorporating spatio-temporal elements and by the use of data-driven modeling techniques such as artificial neural networks.

To fix terminology that will be used throughout the review we define mechanistic and empirical models. Mechanistic models describe how the behavior of a physiological system emerges from the interactions of its constituent parts. Mechanistic models provide an understanding of the system’s internal workings (even when not directly observable) and can predict the system-level behavioral response to changes in its internal parts (e.g., changing a muscle’s contractility). Typically, however, the underlying physiological mechanisms are unknown, and we model only the observable behavior of the system instead of its constituent parts. These so-called phenomenological or empirical models give mathematical descriptions only of the correlations between observable variables. While empirical models can help us systematize the observable behavior of a system, they are constrained to variables that are experimentally accessible. Mechanistic descriptions of each LUT component are preferable for a comprehensive LUT model since explicitly describing each constituent part would create a common basis for different model components to communicate with each other. We may aim for use of mechanistic models to represent all aspects of LUT physiology but turn to the empirical models when a direct mechanism is unknown, as is common for models of LUT neural control.

## 2 | MATERIALS AND METHODS

Model references were found using keyword searches on Web of Science and PubMed related to the individual components of the LUT and mathematical models. References with models that capture connections between at least two LUT tissue-level components were retained and categorized as either a lumped or spatio-temporal model. Lumped models were prioritized for inclusion in the paper, whereas spatio-temporal models were evaluated in terms of how they may provide a more meaningful description of LUT physiology. Seminal models of LUT physiology were also included in our reference pool.

In an effort to promote model credibility, accessibility and interoperability, references were also evaluated according to the Ten Simple Rules (TSR).^9^ This was an effort to assess what rules were typically followed before the publication codifying the TSR. We chose to highlight the following “Five simplest rules” (indicating what percentage of sources satisfied which criteria) which are reflective of what we feel are the minimum requirements to help promote reproducibility:

- Define clearly the context of the model—domain of intended use, clear prediction metrics (83.33%).
- Use contextually appropriate data for validation (4.17%).
- List assumptions and limitations of the model (75.00%).
- Version control using online repositories (0.00%).
- Model is documented appropriately with list of variables, parameters, values, and initial conditions (70.83%).

References with models that met at least one of the five rules were included in the chord diagram (Figure 5).

## 3 | BLADDER

The bladder is a hollow organ that stores urine and periodically empties through the urethral outlet. Mathematical modeling of the bladder involves two distinct but interacting themes: fluid flow in and out (fluid dynamics) and behavior of the bladder walls during filling and voiding (biomechanics).^10^

The most common method for modeling the fluid dynamics of the bladder utilizes a lumped model that considers how its fluid volume *V*_*B*_ changes with time.^11–15^ From the principle of conservation of mass and fixed fluid density, we obtain the relation

(1) $$\frac{dV_{B}}{dt}=Q_{in\;}-Q_{out},$$

where *Q*_*in*_ denotes the net flow rate of fluid into the bladder (either due to the inflow of urine from the kidneys or artificially infused fluid by experiments) and *Q*_*out*_ denotes the flow rate of urine from the bladder into the urethra. A model for *Q*_*in*_ is proposed in Bastiaanssen et al.,^11^ which assumes that the peristaltic inflow of urine can be blocked by high pressure in the bladder:

(2) $$Q_{in}=\{\begin{array}{ll} C_{Q_{in}}, & \;if\;P_{B}<P_{\theta }\\ 0, & \;if\;P_{B}\geq P_{\theta } \end{array},$$

where $C_{Q_{in}}$ is a given constant flow and *P*_θ_ is a pressure threshold. The mathematical description of *Q*_*out*_ is similar and will be discussed in Section 4, Equation (5).

The biomechanics of the bladder is concerned with the visco-elastic properties of its walls. Intuitively, as the bladder distends during filling, tension is generated in its walls. The standard linear solid model (SLS) represents this phenomenon via combinations of springs and dashpots.^16^ Different SLS models and variations, including the addition of an active element that models detrusor contraction, have been used in modeling the bladder.^11–13,17,18^ The biomechanics of the bladder is often simplified by representing the bladder as a spherical shell of thickness *h*_*B*_, internal radius *R*_*B*_, and internal fluid volume *V*_*B*_,^11–15,19^ as illustrated in Figure 2. This assumption has a very important implication: the validity of Laplace law that states that the tension *T*_*B*_ of the bladder walls is proportional to the transmural pressure difference Δ_*trans*,*B*_ = *P*_*B*_ – *P*_*abd*_, where *P*_*B*_ and *P*_*abd*_ represent the pressures in the bladder and abdomen, respectively (see e.g., Eq. 12 in Perez et al.^17^). Explicitly,

(3) $$T_{B}=\frac{R_{B}\Delta_{\text{trans},B}}{2h_{B}}.$$

Laplace law assumes that the circumferential tension does not vary across the wall thickness, which is an appropriate assumption only when the shell is sufficiently thin (*h*_*B*_ ≪ *R*_*B*_). Laplace law establishes a link between the fluid dynamics and biomechanics of the bladder, as it is an algebraic relation between fluid pressure and tension on the bladder walls. Indeed, the circumferential stretch of the bladder as urine flows in produces a tension increase in its walls. This tension increase is then related to the pressure inside the bladder via Equation (3).

**FIGURE 1:**
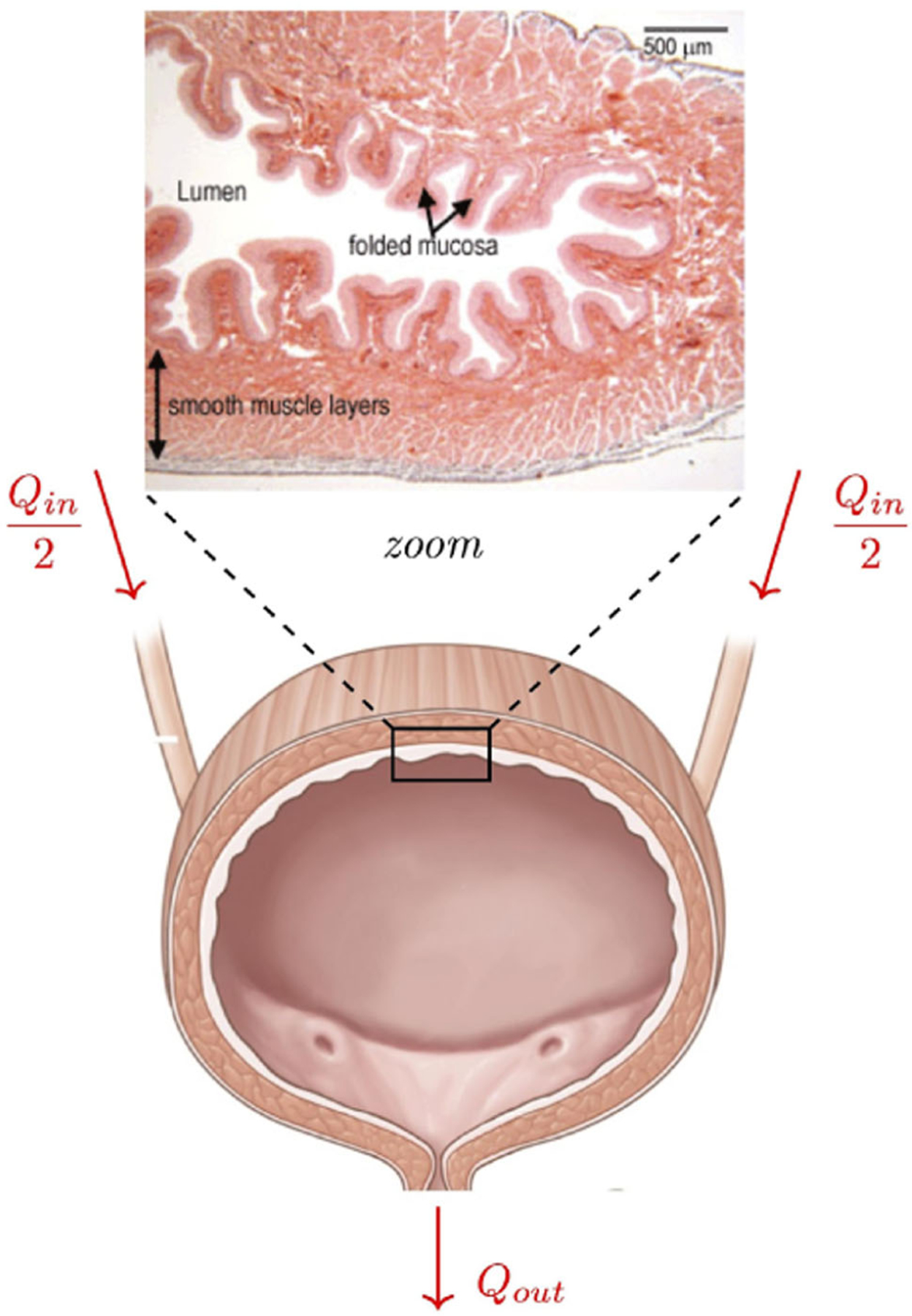
Physiological organization of urine flow from the kidneys into the bladder (*Q*_*in*_) and the flow from the bladder into the urethra (*Q*_*out*_). The geometry of the mucosa of the bladder wall unfolds during volume accumulation, which keeps pressure low during the bladder filling phase. Credit for bladder figure: National Institute of Diabetes and Digestive and Kidney Diseases, National Institutes of Health. Credit for bladder wall figure: The Histology Guide (https://www.histology.leeds.ac.uk).

**FIGURE 2:**
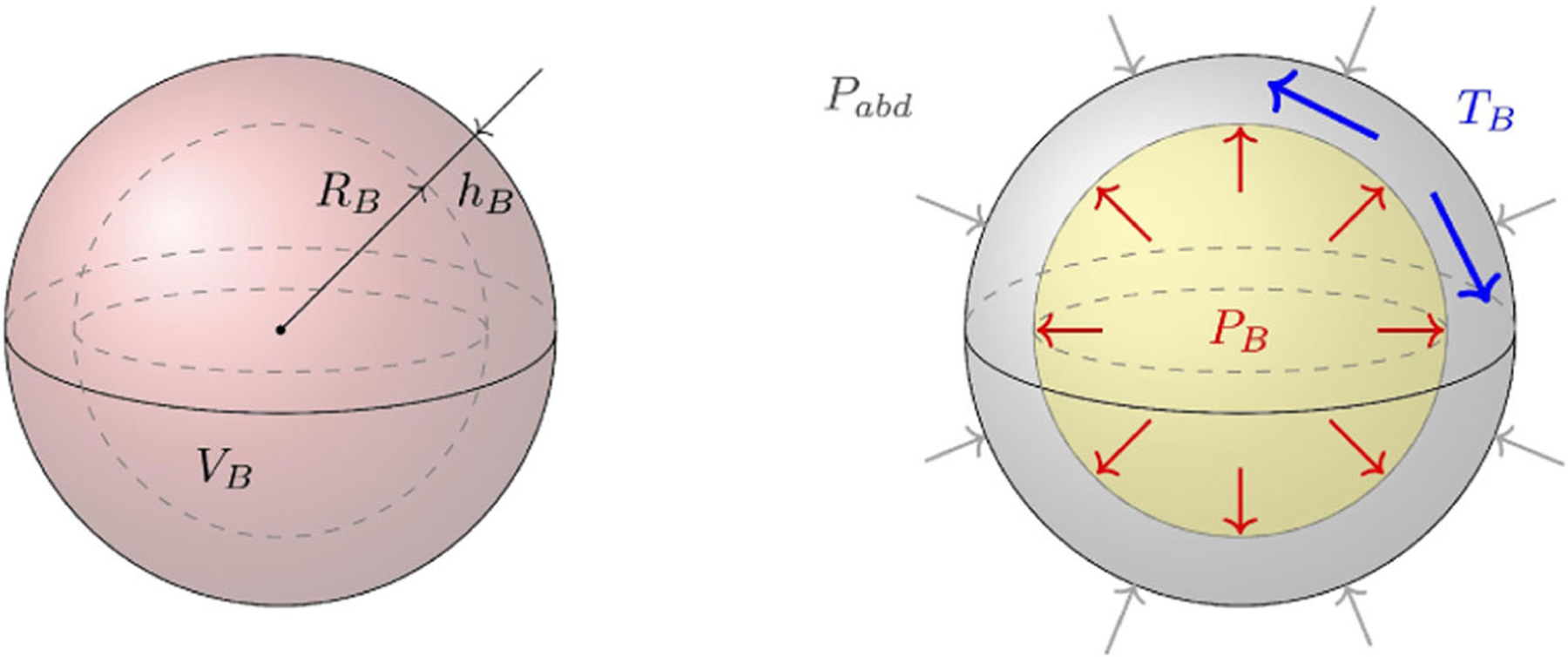
Tension development of an expanding spherical bladder. On the left, a bladder is represented as a spherical shell of volume *V*_*B*_ , radius *R*_*B*_, and thickness *h*_*B*_. On the right, the actions of the abdominal pressure *P*_*abd*_, the fluid pressure *P*_*B*_, and the circumferential tension *T*_*B*_ on the bladder are illustrated. As fluid flows into the bladder and the fluid pressure rises above the abdominal pressure, a circumferential tension is experienced by the bladder wall.

The “unfolding” of the bladder, that is, the flattening of internal rugae during filling,^11,19^ poses a challenge to the spherical assumption discussed above. As the volume of the bladder drops during voiding, its walls fold inward, and the geometry deviates from spherical. During the early stages of the filling phase, the bladder unfolds (Figure 1) to accommodate incoming fluid without pressure or tension being generated. For this reason, Griffiths^19^ considers the spherical assumption valid in humans only when the bladder volume is above 100 ml. This limitation shows that a more detailed study of the bladder geometry is necessary if one wants to understand its behavior during low-volume states, as in the early filling phase and the late voiding phase.

To address more complicated geometries, spatio-temporal models have been used to model the bladder.^7,8,10^ Nonspherical geometries have been investigated by Damaser and Lehman,^20^ where volume-pressure curves were studied for different bladder geometries that indicate bladder fullness cannot be determined from bladder pressure alone.^20^ Tziannaros and colleagues^7,8^ created a model for the collapsing bladder based on the Navier–Stokes equation, providing a detailed model of the fluid dynamics of the bladder during voiding. For example, this model can detect regions of slow flow in the bladder. The authors point out that stagnation may occur in these regions, and thus they are potentially unhealthy regarding urine evacuation.^7^

## 4 | URETHRA

The urethra is a duct that provides a path for urine in the bladder to flow out of the body. The original idealized model for the urethra is the fluid flow through a rigid tube. Under the assumptions that the flow is fully-developed (steady-state), laminar (not turbulent), Newtonian, and through a rigid tube of circular cross-section, the following relation was derived from first principles by Hagen and Poiseuille nearly 200 years ago^21^:

(4) $$Q=\frac{\pi R^{4}\Delta P}{8\mu L},$$

where *Q* is the volumetric flow rate, Δ*P* is the pressure drop between the inlet and outlet of the tube, *R* and *L* represent the inner radius and length of the tube, and *μ* is the fluid viscosity. However, the more realistic case evaluates urethral flow as flow through a collapsible tube.^22–24^ The shape and structure of the collapsible tube is tightly coupled with the fluid flow.^25^ In the case of the urethra, flow is determined by the so-called “flow controlling zone” around the location of the external urethral sphincter.^22–24,26^ From Bernoulli’s theorem and the continuity equation, flow can be described as^19,26^:

(5) $$Q=\{\begin{array}{ll} \alpha {\left(P_{B}-P_{C}\right)}^{\frac{1}{2}}, & \;if\;P_{B}\geq P_{C}\\ 0, & \;if\;P_{B}<P_{C} \end{array},$$

where *P*_*C*_ is a cutoff pressure that determines whether the urethra is opened or closed, *P*_*B*_ is bladder pressure, and *α* is a constant related to the cross-sectional area of the fully relaxed urethra.^26^ In the most idealized models, both *α* and *P*_*C*_ are constant. More realistic models have variable *P*_*C*_ that depends on the ability of the urethra to collapse preventing fluid flow.^27^

The shape and structure of the urethra varies along its length such that consideration of position (through a spatio-temporal model) may provide a more detailed analysis of flow.^22–25^ In the case of the urethra, for a one-dimensional (1-D) unsteady flow model, the balance of linear momentum yields the equation of motion

(6) $$-\frac{1}{\rho }\frac{\partial P_{U}}{\partial x}=\frac{\partial u}{\partial t}+u\frac{\partial u}{\partial t},$$

where *ρ* is the mass density, *P*_*U*_ is the fluid pressure in the urethra, *u* is the fluid velocity, *x* is the axial position, and *t* is time. From this equation, we see how changes in pressure produce changes in fluid velocity. From the balance of mass, we obtain the equation

(7) $$\frac{\partial A}{\partial t}+\frac{\partial }{\partial x}(Au)=0,$$

where changes in cross-sectional area *A* are coupled to changes in fluid velocity. Finally, Equations (6) and (7) are linked by an empirical tube law, for example:

(8) $$\frac{-P_{\text{trans},U}}{K_{P}}={\left(\frac{A}{A_{0}}\right)}^{\frac{-3}{2}}-1,$$

where Δ*P*_*trans*,*U*_ = *P*_*U*_ − *P*_*abd*_ is the transmural pressure drop (the difference between the pressure in the urethra and the abdominal pressure), *K*_*P*_ is proportional to the bending stiffness of the tube wall, and *A*_0_ the cross-sectional area when Δ*P*_*trans*_ = 0.

Of course, the urethra does not have a simple tube-like geometry and interacts biomechanically with both the internal and external urethral sphincters differently across its length and in three dimensions (3-D). In exploring more complex urethral flow regimes, 2- and 3-D models were designed for which analytical and numerical results have been developed.^8,10,28^ In more recent years, detailed anatomically-accurate representations of the urethra have been created based on histological and morphometric studies or using magnetic resonance imaging (MRI). For example, experimental and structural studies of the male urethra have been combined with 3-D finite element models to characterize urethral occlusions using artificial devices to provide urinary continence^29,30^; in addition, MRI studies of the female urethra have been combined with computationally intensive finite element models to simulate how contraction of striated and smooth muscles contribute to the urethral closure pressure (*P*_*C*_ ).^31^ Allometric analyses of LUT function across animals with body mass ranging from 0.03 to 8000kg find urethral length, diameter, bladder capacity, and steady-state flow rate scale with body mass, but bladder pressure and duration of urination are relatively insensitive to body size.^32^ Moreover, such analyses suggest that small-rodent studies guiding models of the human LUT may need to be adapted to put less attention on viscous and capillary forces and more attention on the role of hydrostatic pressure.

## 5 | NEURAL CONTROL

While the bladder and urethra are usually idealized as a sphere and a collapsible tube, the neural analog is electric circuitry. These circuits transduce mechanical work into voltage (for sensory neurons) or vice versa (for motor neurons). Neural models come in a range of modalities - some use empirically linked circuit outputs to LUT fluid dynamics,^33–35^ some focus on the behavior of specific neurons,^36^ and others create elaborate controllers to mimic the central integration of signals.^11,13,17,18^ In empirical models,^33–35^ pressure is the typical driving mechanical stimulus used because it is highly correlated with distension (the putative driver of afferent signaling)^37^ in most physiological states and is readily accessible experimentally. The le Feber approach utilizes a piecewise-defined function to model changes in pressure and pudendal sensory nerve activity based on the onset and duration of externally infused urethral fluid - an approach that generates excellent fits to their original data but limits its predictive validity beyond the protocol in which it was fit.^34^ To facilitate protocol-independent prediction, Danziger and Grill proposed a more general, autonomous model of the sensory pudendal nerve that uses pressure *P*(*t*), its time rate of change, and the nerve activity *s* to approximate the time rate of change of neural activity^35^:

(9) $$\frac{ds}{dt}=k\left(f_{0}(P(t))+f_{1}\left(\frac{dP(t)}{dt}\right)-s\right)s,$$

where *k* is a time constant, and *t* is time. The transduction functions *f*_0_ and *f*_1_ map *P*(*t*) and $\frac{dP(t)}{dt}$ respectively into the rate of change in neural activity.^35^

The pudendal nerve was likely the first focus of bulk nerve modeling because its neuroanatomical organization permits physical separation of the sensory and motor fibers.^35,38^ This allows isolation of these fiber types for easier model validation via unambiguous electrophysio-logical recordings. This is a luxury not afforded to the pelvic nerve where mixed fiber types travel in the same fascicles. Attempts to dissociate the afferent and efferent activity of the pelvic nerve through different assumptions or experimental design are difficult to validate - though linear separability of mixed nerve activity has been postulated.^33^

**FIGURE 3:**
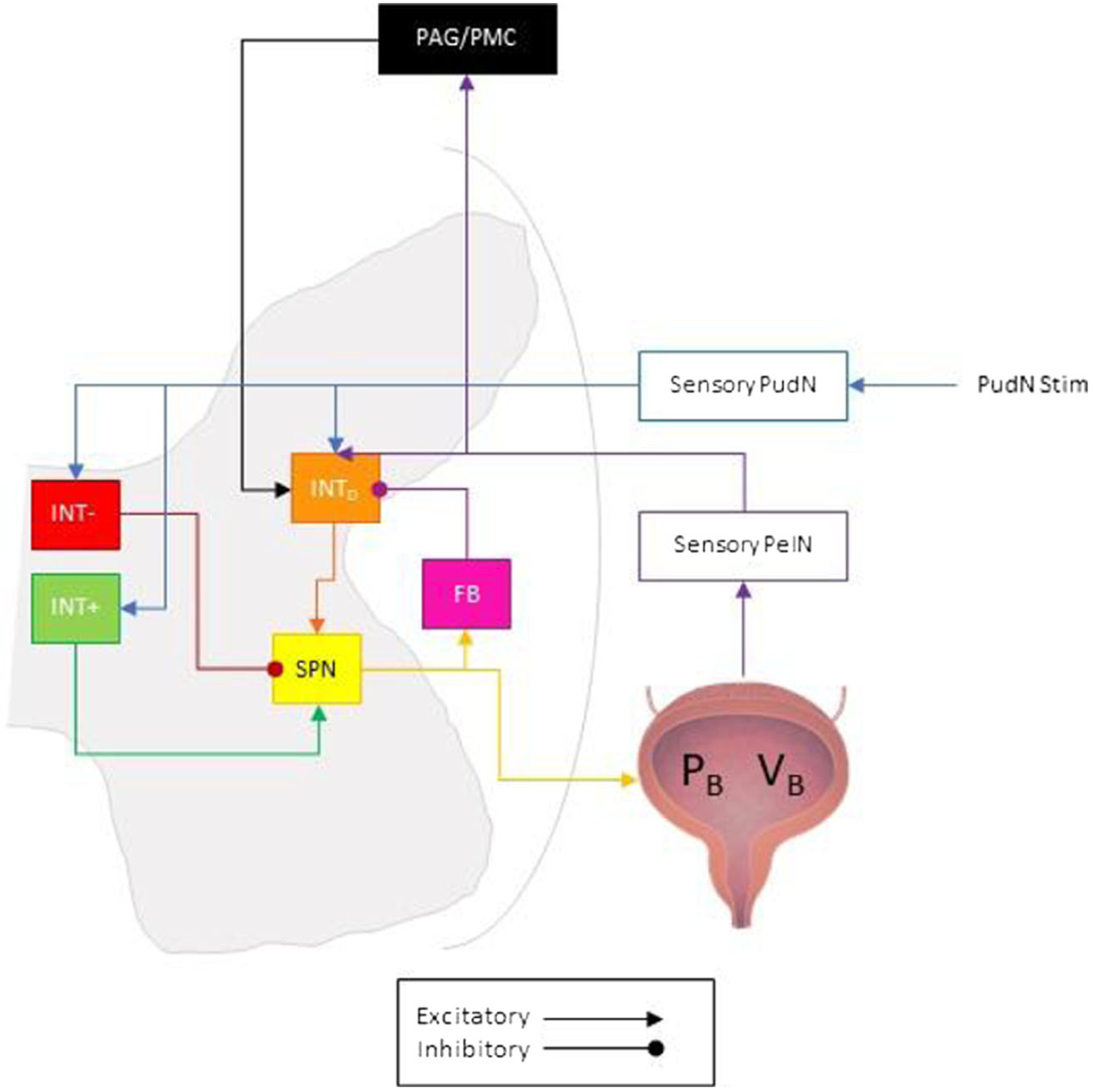
The neural network of linear integrate and fire neurons describing the pudendo-vesical reflex as proposed by McGee and Grill.^39^ Bladder pressure (*P*_*B*_) was simulated as a function of bladder volume (*V*_*B*_) and the nonmechanistic polynomial fit of the approximate sacral parasympathetic nerve (SPN) firing rate in response to experimental bladder pressure. The afferent pelvic nerve (PelN) firing rate was iteratively determined from *P*_*B*_ and a nonmechanistic polynomial fit of low threshold PelN response to experimental bladder pressure. The model includes a supraspinal switching node (illustrated as the periaqueductal gray (PAG) and pontine micturition center (PMC)) dependent on PelN firing rate and *V*_*B*_. Excitatory postsynaptic synapses were modeled to reflect glutamatergic receptors, whereas inhibitory postsynaptic synapses were modeled to reflect *GABA*_*A*_ (γ-Aminobutyric acid) receptors, both of which are common to spinal cord neurons. Feedback interneuron (FB), dorsal interneuron (INT_D_), medial excitatory (INT+) and inhibitory (INT−) interneurons. (Bladder in Figure: DataBase Center for Life Science (DBCLS) Published May 2014. Accessed January 20, 2022. http://dbcls.rois.ac.jp/ Adapted.)

Models of individual neurons are sparse in LUT literature, and each uses different approaches. Linear integrate and fire (LIF) neurons were used to construct the neural network associated with the pudendo-vesical reflex in Figure 3.^39^ The McGee model provides a plausible heuristic for the integration of neural activity in the spinal cord, which was previously modeled with neurophysiologically uninformed control systems.^13,18,40^ Similar to the Danziger model, the McGee model uses pressure directly to compute its predictions of neural activation, allowing it to be combined straightforwardly with previously described biomechanics models. Conversely, Hodgkin–Huxley model neurons operate at cellular and intracellular scales to evaluate ion channel dynamics, often impairing computational tractability with organ-scale models.^36^

**FIGURE 4:**
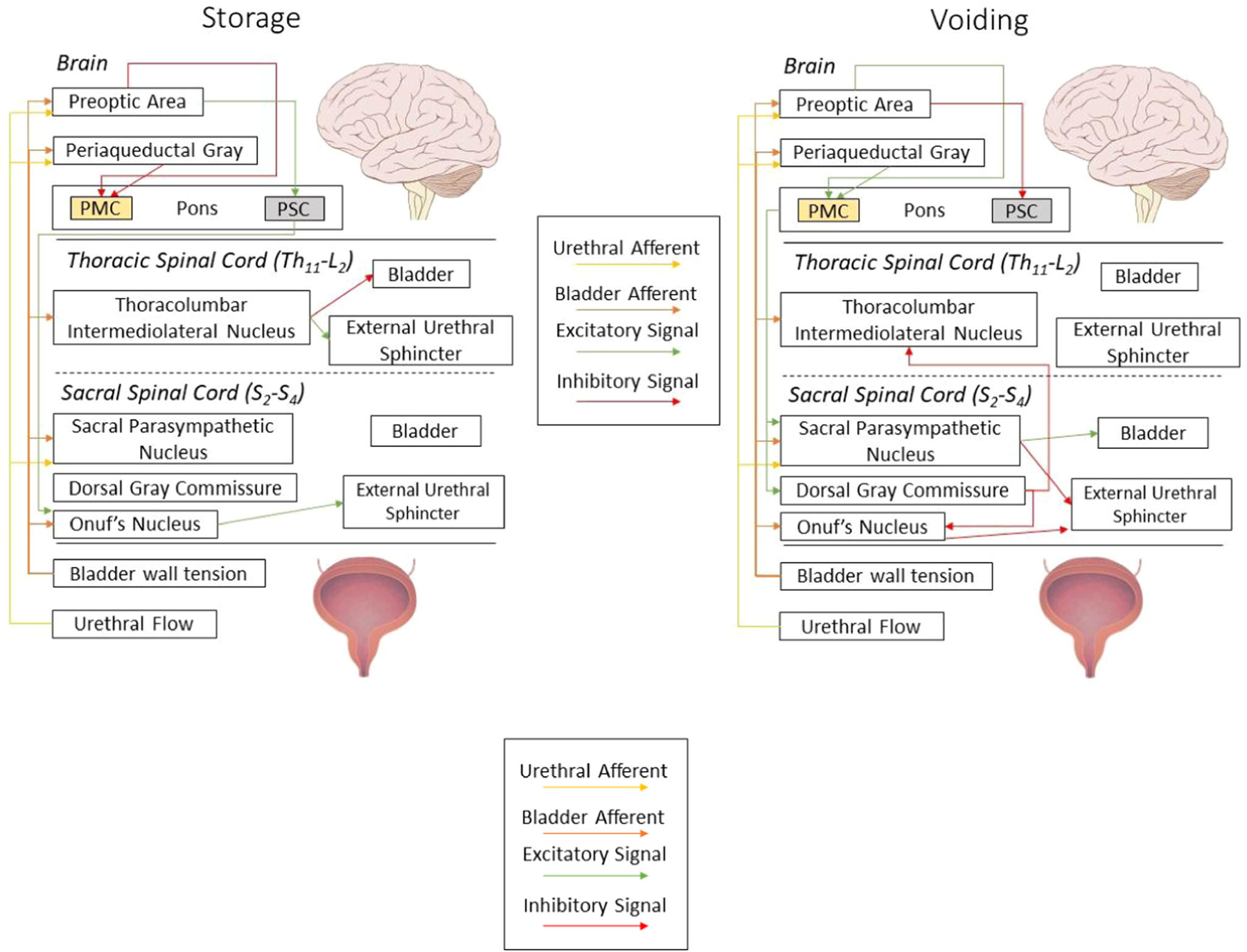
The general layout of a control model, adapted from Paya et al.^13^ Feedback from the mechanical model is sent through a network of CNS controllers that operate as threshold-gated switches. The switches communicate to determine if a change in state (storage, Left or voiding, Right) is required. The “switch” from storage to voiding depends on the voluntary initiation from the Preoptic area of the brain. The pons houses the pontine micturition center (PMC) and the pontine storage center (PSC) corresponding to the M and L regions of the pons. During storage, the PMC is inhibited, with an active PSC which stimulates Onuf’s Nucleus to activate the external urethral sphincter (EUS). The thoracolumbar intermediolateral nucleus further activates the EUS and relaxes the bladder muscle. While voiding, the cortical areas activate the PMC which stimulates the sacral parasympathetic nucleus and the dorsal gray commissure. The dorsal gray commissure inhibits the thoracolumbar nucleus and Onuf’s nucleus. The sacral parasympathetic nucleus activates the contraction of the bladder and the relaxation of the EUS. (Brain: Lynch PJ, Jaffe CC, Yale University School of Medicine, Center for Advanced Instructional Media, 1987–2000. Published December 23, 2006. Accessed January 20, 2022. http://patricklynch.net Adapted. Bladder in Figure: DataBase Center for Life Science (DBCLS) Published May 2014. Accessed January 20, 2022. http://dbcls.rois.ac.jp/ Adapted.)

The central coordination of neural signals from the LUT has often been described as a switching mechanism to change between phases of filling and voiding.^40,41^ In each phase, for instance, the bladder and sphincter muscles have coordinated, reciprocal activation patterns to promote the current phase of the system. Consequently, modelers have used this analogy to create control models utilizing threshold-gated circuits to switch between filling and voiding. What started out as a single switch controller,^40^ has evolved to better represent (at least qualitatively) the neuroanatomical organization of LUT controllers in the central nervous system (Figure 4).^13^ The de Groat model used a different approach (similar to Bastiaanssen et al.^42^), where more connections were used and specific details about individual neurons were incorporated into their design (e.g., in vivo data were used to tune the weights of interactions between specific neurons).^41^ The advantage to the de Groat model compared to the other control models,^13,18^ lies in the modularity of the model’s neurons; however the model is more focused on conceptually demonstrating that a switching circuit could be constructed from known neuron types rather than elaborating a physiologically accurate connectome. Despite the ability of control models to produce nominal behavioral profiles accurately (e.g., voiding pressure traces), there are no physiological quantities with which models of disease and therapies can interface. Though therapeutic modeling is not currently prevalent in LUT literature, an important step towards ensuring that it can be a possibility is to use quantitative physiological inputs and outputs derived from mechanistic representations of the central neural connections. For this reason, a shift toward mechanistic representations and away from control models is warranted.

## 6 | CONNECTIONS

**FIGURE 5:**
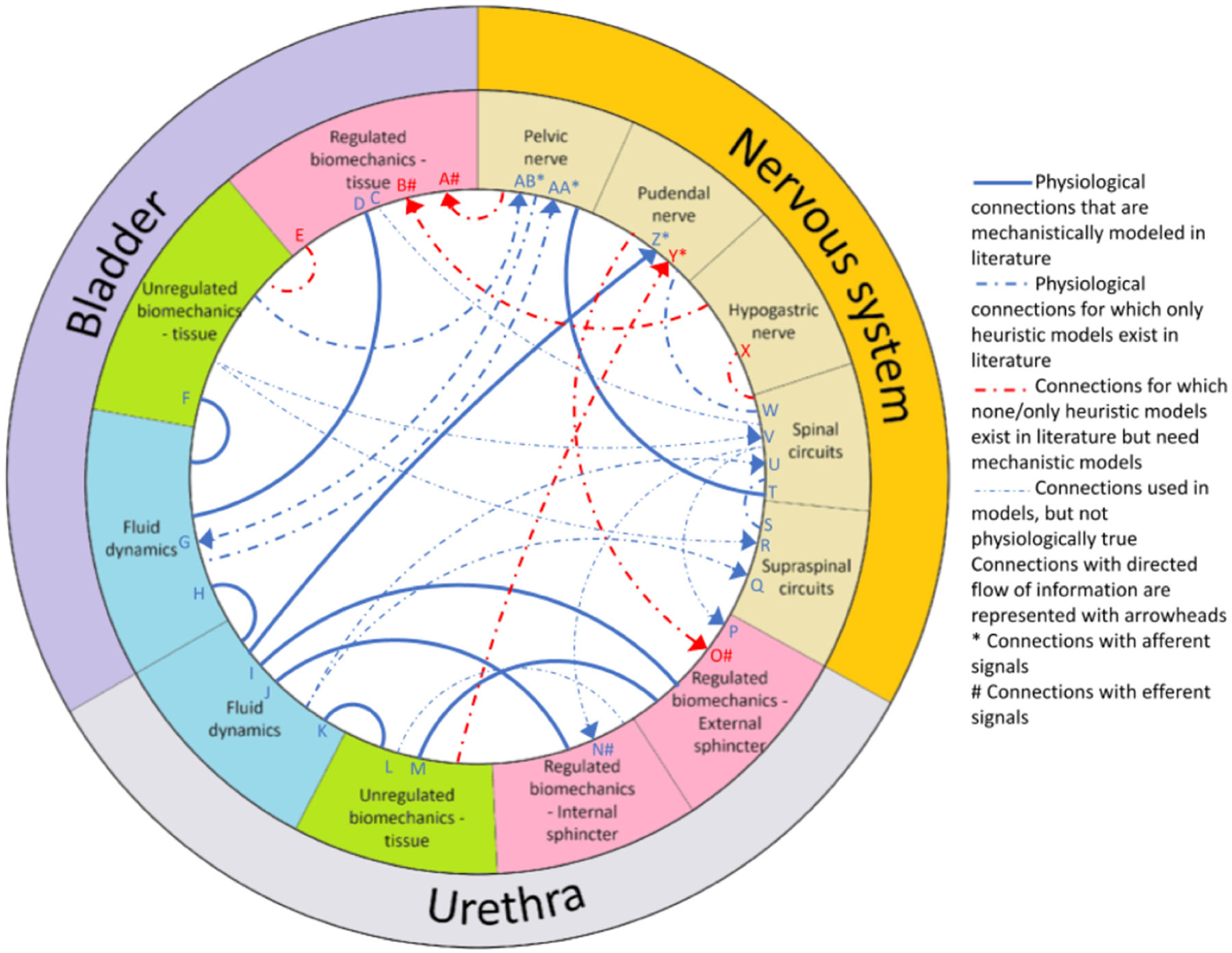
Models to date that represent connections between at least two LUT tissue-level components. Regulated biomechanics refers to tissue controlled directly by motor commands from the nervous system (e.g., contracting muscle), whereas unregulated biomechanics refers to forces generated by passive tissue properties without neural intervention (e.g., elasticity). Fluid dynamics and biomechanical forces act reciprocally to determine their behaviors, and so are rendered without arrows. Neural components have characteristic directionality (e.g., commands travel from the CNS to peripheral muscles and sensory signals travel from the peripheral tissue to the CNS), and so are rendered with arrows. The line styles distinguish between three types of component models: (Solid) Those motivated by known physiological interactions, (Dashed Blue), those using heuristics to represent known physiological interactions, (Dotted Blue), those positing direct connections between components know to be aphysiological, typically to reduce overall model complexity, and (Red) those connections for which mechanistic models are likely to be critical for establishing a comprehensive LUT model but for which we currently only have heuristics or no mathematical descriptions at all. A,^14^ C,^13,17,18^ D,^8,11,13,17,18,33^ E,^11,13,17,18,37^ F,^8,11,13,17,18,37,40,44,45^ G,^39^ H,^11,13,17–19^ I,^13,17^ J,^13,17^ K,^10,13,15,17,18^ L,^13,17^ M,^11,13,17^ N#,^13,17^ O#,^14^ P,^13,15,17,18^ Q,^13,15,17^ R,^13,15,17,40^ S,^13,17,18,39,41^ T,^39^ U,^13,15,17,18^ V,^13,15,17,18^ W,^39^ Z*,^35^ AA*,^33,37,46,47^ AB*.^37,46^

Due to its complexity, the development of a functionally predictive systems physiology model of the LUT will likely have to be a modular process in which independently designed models of individual components, such as the ones surveyed thus far, will fit together in a unified model. Toward this goal, a diagram of existing LUT component models and their interconnectivity can clarify the state of the field, establish a common conceptual framework, help investigators put their models in the context of larger modeling efforts, encourage interoperability, and identify major knowledge gaps.^43^ We developed a chord diagram in Figure 5 to synthesize LUT modeling efforts and the connections between them (arrows). Figure 5 highlights a lack of physiologically-based mathematical descriptions of neural connections, and while some heuristic replacements for these have been proposed (blue dashed), it is crucial going forward that the inputs and outputs of each model represent real physiological quantities if it is to be integrated into the broader LUT modeling endeavor. The diagram also makes evident connections with no available mechanistic model (red dashed), but that will likely be indispensable for a full functionally predictive LUT model, making them high priority for future work.

There are technical considerations that might make certain models more amenable for a modular and collaborative project of this sort. Perhaps the most fundamental aspect of LUT function is the fact that it occurs in cycles, each consisting of two phases: filling and voiding. For different models to operate together, their dynamics should be able to reproduce the cyclic behavior of the LUT. Lumped models have so far been more successful in simulating cycles, as in Paya et al.^13^ and Perez et al.^17^ On the other hand, spatio-temporal models, despite the precision with which they can represent certain phenomena, tend to fail to simulate both phases. For example, the spatio-temporal model of the bladder proposed by Tziannaros et al.^8^ can predict with great detail the fluid dynamics of the voiding phase, while the filling phase is absent from the model.^37,44–47^

A field-wide LUT modeling effort across multiple independent investigators necessitates a set of standard good practices to help facilitate the integration of new models into the modular framework, such as those suggested in Erdemir et al.^9^ to improve credibility, accessibility and inter-operability. Adherence to the TSR, especially open-source test data and online repositories of model code, is fundamental to the success of a collaborative and long-term project.

To progress towards a predictive organ-scale model, models of neural interactions with LUT organs will need to be more physiologically motivated and conform better to good modeling practices in the future, mirroring work on bladder biomechanics models. But a significant amount of modeling efforts and physiological exploration are still necessary to achieve this goal. There also is motivation to explore smaller physiological scales which will be a crucial step towards predicting the propagation of cellular-level effects from, for example, pharmacological interventions.^48–50^ It is important that these smaller-scale models consider how they might connect with the larger components for inclusion into the comprehensive model by having a unifying physiological interaction (such as tension^49^) between anatomical levels.

## 7 | CONCLUSION

Even though a comprehensive, functionally predictive model of the LUT is presently unavailable, a significant body of work modeling different components of the LUT has been developed over several decades. We have surveyed models of the three main components of the LUT: bladder, urethra, and neural control. In the case of the bladder and the urethra, we have privileged lumped models as their simplicity makes them the best option, at least initially, for incorporation into a unified model. The models we have reviewed should not be regarded as finalized projects, but rather as stepping stones for the next challenges that lie ahead. From our perspective, the most urgent of which is to create new mathematical descriptions of the neural control of the LUT with a view towards developing a comprehensive model of LUT function. It appears to us that the field is ready to systematically undertake this challenge.

## Data Availability

Data sharing is not applicable to this article as no datasets were generated or analyzed during the current study.
